# A Recursive Model of the Spread of COVID-19: Modelling Study

**DOI:** 10.2196/21468

**Published:** 2021-04-19

**Authors:** Sergey O Ilyin

**Affiliations:** 1 AV Topchiev Institute of Petrochemical Synthesis, Russian Academy of Sciences Moscow Russian Federation

**Keywords:** epidemiology, COVID-19, model, modelling, prediction, spread, infection, effective, contagious, transmission

## Abstract

**Background:**

The major medical and social challenge of the 21st century is COVID-19, caused by the novel coronavirus SARS-CoV-2. Critical issues include the rate at which the coronavirus spreads and the effect of quarantine measures and population vaccination on this rate. Knowledge of the laws of the spread of COVID-19 will enable assessment of the effectiveness and reasonableness of the quarantine measures used, as well as determination of the necessary level of vaccination needed to overcome this crisis.

**Objective:**

This study aims to establish the laws of the spread of COVID-19 and to use them to develop a mathematical model to predict changes in the number of active cases over time, possible human losses, and the rate of recovery of patients, to make informed decisions about the number of necessary beds in hospitals, the introduction and type of quarantine measures, and the required threshold of vaccination of the population.

**Methods:**

This study analyzed the onset of COVID-19 spread in countries such as China, Italy, Spain, the United States, the United Kingdom, Japan, France, and Germany based on publicly available statistical data. The change in the number of COVID-19 cases, deaths, and recovered persons over time was examined, considering the possible introduction of quarantine measures and isolation of infected people in these countries. Based on the data, the virus transmissibility and the average duration of the disease at different stages were evaluated, and a model based on the principle of recursion was developed. Its key features are the separation of active (nonisolated) infected persons into a distinct category and the prediction of their number based on the average duration of the disease in the inactive phase and the concentration of these persons in the population in the preceding days.

**Results:**

Specific values for SARS-CoV-2 transmissibility and COVID-19 duration were estimated for different countries. In China, the viral transmissibility was 3.12 before quarantine measures were implemented and 0.36 after these measures were lifted. For the other countries, the viral transmissibility was 2.28-2.76 initially, and it then decreased to 0.87-1.29 as a result of quarantine measures. Therefore, it can be expected that the spread of SARS-CoV-2 will be suppressed if 56%-64% of the total population becomes vaccinated or survives COVID-19.

**Conclusions:**

The quarantine measures adopted in most countries are too weak compared to those previously used in China. Therefore, it is not expected that the spread of COVID-19 will stop and the disease will cease to exist naturally or owing to quarantine measures. Active vaccination of the population is needed to prevent the spread of COVID-19. Furthermore, the required specific percentage of vaccinated individuals depends on the magnitude of viral transmissibility, which can be evaluated using the proposed model and statistical data for the country of interest.

## Introduction

The first mathematical models to predict the development of infectious diseases were used in the early 20th century [1,2]. In 1927, Kermack and McKendrick [3] proposed the use of differential equations for calculations, dividing the human population into people susceptible to disease (S) and those who had already recovered (R). The susceptible persons became infected (I) at some rate of transmission and then recovered at a different rate. Their model became known by the acronym SIR, which means that the model simultaneously calculates the number of susceptible, infected, and recovered persons. This model served as a basis for the development of subsequent models—by modifying the equations and adding to the calculation other persons not belonging to the three specified basic categories, which allowed consideration of the features of particular diseases. Since then, various models have been created that consider the possibility of re-infection (SIS model) [4] and death (SIRD model) [5], the existence of an incubation period (SEIR model) [6], and temporary immunity of infants (MSIR model) [7], among others.

When a new infection appears, neither the set of population categories to be considered in the model nor the rate of transition of people from one category to another is known. Current information about the features of the COVID-19 infection caused by the novel coronavirus (SARS-CoV-2) and the manner in which people perceive it and act should serve as a basis for building a model to describe the spread of this virus. These features can be described as follows: first, the presence of a long incubation period, during which the infected persons are contagious to others, and second, the isolation of discovered infected persons, which as a result become conditionally noncontagious. The combination of these two factors makes this novel coronavirus infection unique. In general, the opposite is true—infected people are not dangerous to others during the incubation period and become contagious after its expiry. For this reason, a new model that considers these circumstances is needed to predict the spread of COVID-19. However, the duration of the immunity produced after recovery from COVID-19 is currently unknown. In addition, there is also very little information available to accurately calculate the rate of recovery among patients with COVID-19: a small percentage of the population recovers within just a week after contracting infection, whereas the majority of people experience the illness for a long time. Therefore, the proposed model cannot be final, but it is necessary for forecasting and management decisions.

## Methods

The model for COVID-19 spread is based on a set of parameters whose values are unique for each country due to differences in population density, human behavior, date of virus penetration, and government actions. The set includes the following parameters:

- *d*_0_ is the date of the initiation of the epidemic; it is not the date of detection of the first infected person but the date of appearance of the first undetected (or detected too late) person.
- *d*_1_, *d*_2_, and *d*_3_ are dates of change in the behavior of the population, for example, due to the awareness of the reality of what is happening and the introduction of quarantine and its tightening.
- *t*_D_ is the average time from infection to isolation of the infected person, which is equal to the incubation period assumed to be 6 days (ranging from 5.2 to 6.4 days according to different sources [8,9]); theoretically, this parameter can be reduced by testing of the entire population, but it is feasible only for small communities.
- *R*_0_, *R*_1_, *R*_2_, and *R*_3_ are the viral transmissibilities that are equal to the average number of people who will be infected by one person before he or she is isolated and depend on the behavior of the population at different stages of the epidemic; when *R* is less than 1.0, the epidemic fades, and vice versa.
- *r*_0_, *r*_1_, *r*_2_, and *r*_3_ are the reduced viral transmissibilities that are equal to the average number of people who will be infected by one person per day: *r* = *R*/*t*_D_; to suppress the spread of COVID-19, *r* should be less than 0.167.

The evaluation of the spread of the virus is based on the calculation of the following data:

- *N*_D_(*d*_i_) is the number of infected persons detected on *d_i_* date, which equals the total number of infected persons 6 days earlier:

*N*_D_(*d*_i_) = *N*_T_(*d*_i_-*t*_D_)

- *N*_T_(*d*_i_) is the total number of infected persons on date *d*_i_, which is the sum of the total number of infected persons the day before and the number of new infected persons that, in turn, is equal to the product of the reduced transmissibility and the number of active infected persons the day before (taking into account that those who have been previously infected cannot be reinfected):

*N*_T_(*d*_i_) = *N*_T_(*d*_i_-1)+*r*_0_×*N*_A_(*d*_i_-1)×[1-*N*_T_(*d*_i_-1)/*N*_P_],

where *N*_P_ is the total population.

In the case of vaccination of the population and considering the temporary nature of the immunity received due to SARS-CoV-2 infection or vaccination, the above expression will be as follows:

*N*_T_(*d*_i_) = *N*_T_(*d*_i_-1)+*r*_0_×*N*_A_(*d*_i_-1)×[1-[*N*_T_(*d*_i_-1)+ *N*_V_(*d*_i_-1)-*N*_T_(*d*_i_-1-*t*_im_)-*N*_V_(*d*_i_-1-*t*_im_)]/*N*_P_],

where *t*_im_ is the average duration of preserving full immunity against the virus after vaccination or disease, whereas *N*_V_(*d*_i_) is the total number of vaccinated persons on date *d_i_* who have not had COVID-19 in the last *t*_im_ days;

- *N*_A_(*d*_i_) is the total number of active (undetected) infected persons on date *d_i_*, which equals the difference between the total number of infected persons and the number of infected persons detected on the same day:

*N*_A_(*d*_i_) = *N*_T_(*d*_i_)-*N*_D_(*d*_i_).

At the start of the epidemic (date *d*_0_), *N*_A_(*d*_0_) = 1, *N*_T_(*d*_0_) = 1, and *N*_D_(*d*_0_) = 0.

Thus, in order to calculate the virus spread dynamics, it is necessary to know the values of only two parameters—*d*_0_ and *r*_0_. In the case of changing the behavior of the population from the date *d*_1_, parameter *r*_0_ changes its value from this date to become *r*_1_. If the behavior changes again, a pair of *d*_2_ and *r*_2_ will appear, and so on.

However, it is more difficult to model human losses correctly. Two more parameters need to be considered:

- *L* is the apparent lethality rate that is equal to the ratio of the number of deaths to the sum of those who died or recovered;
- *t*_L_ is the average time from infection to death.

These two parameters depend on the efficacy of treatment and may vary as physicians gain experience and as hospitals overflow. The number of deaths on date *d*_i_ equals the total number of people infected *t*_L_ days earlier multiplied by the lethality rate:

*N*_L_(*d*_i_) = *N*_T_(*d*_i_-*t*_L_)×*L*

Because of the presence of two parameters (*t*_L_ and *L*) in the equation, which have the same effect on the resulting value, the precision of their evaluation is lower than that for viral transmissibility. It should be understood that the fewer the number of asymptomatic and mild cases of the disease have been detected, the more the lethality rate is overestimated. The average time from infection to death was found to be about 8 days, and this duration will be used to make calculations for all countries.

The situation with predicting the number of recovered persons is even worse due to the appearance of an even greater number of independent parameters:

*N*_R_(*d_i_*) = *N*_T_(*d*_i_-*t*_M_)×*k*_M_+*N*_T_(*d*_i_-*t*_S_)×*k*_S_

where *k*_M_ and *k*_S_ are the shares of mildly and seriously ill patients (*k*_M_+*k*_S_+L = 1), and *t*_M_ and *t*_S_ are the corresponding times from infection to healing:

*k*_M_+*k*_S_+*L *= 1

The model equations are presented in the discrete form (instead of differential one), so that the model can be easily reproduced for calculations in any spreadsheet editor. At first glance, it seems that the model does not take into account the existence of asymptomatic carriers of infection, but this is not true: since the share of asymptomatic carriers in the population does not change over time, their presence is taken into account implicitly by the value of the transmissibility. This model can be denoted by the abbreviation SILRD, which means that it takes into account Susceptible, Infected, Isolated, Recovered, and Dead persons.

## Results

**Figure 1:**
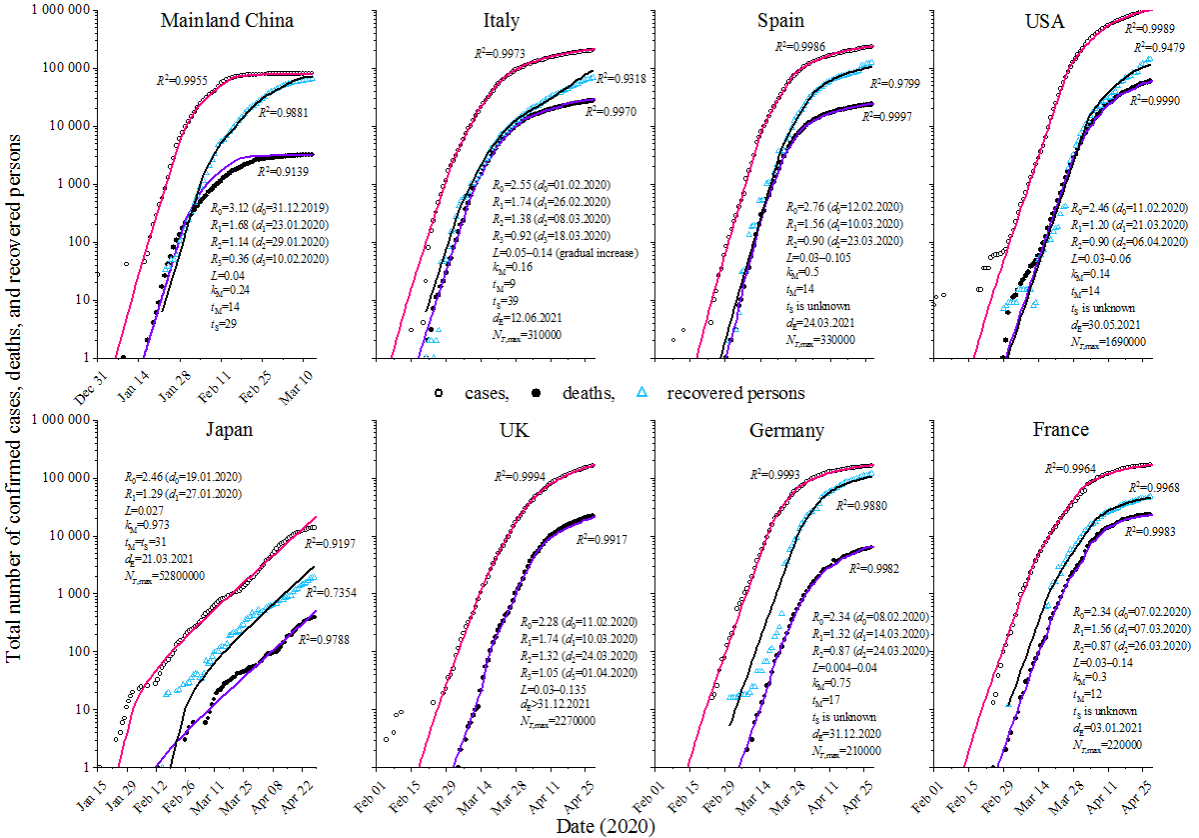
Time dependences of the total number of COVID-19 cases, deaths, and recovered cases. Dots show actual data, whereas lines represent the result of calculations using the model.

Based on historical data on disease development in eight countries (China, Italy, Spain, the United States, the United Kingdom, Japan, France, and Germany [10]), the model was tested (Figure 1) and most of its parameters were found (Table 1). For all the countries, the viral transmissibility at the start of the epidemic was between 2.28 and 3.12. The highest viral transmissibility was found in China, wherein one person infected three others, probably because of higher population density. The introduction and progressive strengthening of quarantine measures resulted in a decrease in the viral transmissibility, which was noticeable 6 days later in the decline in the rate of new cases. All the countries had introduced quarantine measures gradually. The initial restrictions reduced the viral transmissibility to 1.20-1.74, which was not adequate (it was necessary to achieve a transmissibility of less than 1.0), and the virus continued to spread with acceleration. As a result, all the countries, with the exception of Japan, initiated stricter measures, thus reducing the transmissibility to 0.87-1.14. Japan focused on timely detection and isolation of infected persons. It can be concluded that this strategy does not work, as can be observed from the curve of the total number of cases in Japan that alternately slows down and then accelerates again. This is the result of the fact that Japan has been successfully isolating most of the infected persons, but a few infected people remain nonisolated and they can cause another outbreak to occur. By contrast, China further strengthened its containment measures, which resulted in a reduction of the transmissibility to 0.36 and a quick win over the epidemic (in 6 weeks according to the model).

**Table 1:** Parameters identified from the models used in different countries.

Parameter	China	Italy	Spain	USA	Japan	UK	Germany	France
*R*_0_	3.12	2.55	2.76	2.46	2.46	2.28	2.34	2.34
*d*_0_	31.12.19	01.02.20	12.02.20	11.02.20	19.01.20	11.02.20	08.02.20	07.02.20
*R*_1_	1.68	1.74	1.56	1.20	1.29	1.74	1.32	1.56
*d*_1_	23.01.20	26.02.20	10.03.20	21.03.20	27.01.20	10.03.20	14.03.20	07.03.20
*R*_2_	1.14	1.38	0.90	0.90	N/A^a^	1.32	0.87	0.87
*d*_2_	29.01.20	08.03.20	23.03.20	06.04.20	N/A	24.03.20	24.03.20	26.03.20
*R*_3_	0.36	0.92	N/A	N/A	N/A	1.05	N/A	N/A
*d*_3_	10.02.20	18.03.20	N/A	N/A	N/A	01.04.20	N/A	N/A
*L* ^b^	0.04	0.05–0.14	0.03–0.105	0.03–0.06	0.027	0.03–0.135	0.004–0.04	0.03–0.14
*k*_M_	0.24	0.16	0.5	0.14	0.973	N/A	0.75	0.3
*t*_M_	14	9	14	14	31	N/A	17	12
*t*_S_	29	39	unknown	unknown	31	N/A	unknown	unknown
*d*_E_	N/A	12.06.21	24.03.21	30.05.21	21.03.21	>31.12.21	31.12.20	03.01.21
*N*_T,max_	N/A	310000	330000	1690000	52800000	2270000	210000	220000

^a^Not applicable.

^b^If an interval is indicated, it means gradual growth.

## Discussion

On the date on which the analyzed data set ends, all European countries (except the United Kingdom) only managed to reduce the transmissibility slightly below 1.0. From a practical point of view, this means that the number of people falling ill on a daily basis in these countries was gradually decreasing, but it was at such a slow pace that the end date of the epidemic in these countries could not have been before, at best, the end of 2020. In reality, these countries have partially canceled quarantine measures, causing an increase in viral transmissibility and, consequently, a new rise in the number of infected persons and a shift in the date of a possible end of the epidemic to the future. It should be understood that any alleviation of quarantine measures would lead to increased transmissibility and resumption of an accelerated spread of the virus. To prevent this from happening after the quarantine restrictions have been removed, the viral transmissibility must remain below 1.0. By way of example, the original transmissibility was 2.55 in the case of Italy; therefore, it is necessary that 61% of the Italian population be either infected and then recovered (provided the immunity produced is durable and strong) or vaccinated against SARS-CoV-2 so that when quarantine measures are lifted, the transmissibility remains less than 1.0. At the time of writing this manuscript, 0.33% of the Italian population had been infected according to official statistics [10]. Statistics may not take into account asymptomatic and mild cases of the disease, numbers of which may be 4-50 times higher (for the time being, only by rumor) than that of the officially recorded cases. Even if this were true, the percentage of infected and recovered persons is still significantly lower than necessary, and the removal of quarantine measures will inevitably lead to a return of the growth rate of the number of infected people to almost their original level. In other words, the rapid development, production, and subsequent application of a vaccine are vital to overcoming the COVID-19 crisis in the near future.

Thus, the model allows forecasting of the situation development and concluding about the effectiveness of quarantine measures. By way of example, it helps determine the current number of active infected persons (*N*_A_(*d*_i_)), the approximate date of isolation of the last infected person (*d*_E_), and the number of people that could eventually be infected under the current quarantine (*N*_T,max_). According to the calculations, the efforts made by many European countries, the United States, and Japan to stop the spread of COVID-19 are not as effective as those implemented previously in China. Most countries have been able to achieve a daily reduction in the number of infected people, but even in these cases, the viral transmissibility remains high enough, which does not allow the country to overcome the epidemic crisis within a reasonable time. At the same time, suppressing the epidemic, albeit slowly, allows time for vaccine development and launch into mass production.
